# Alpha-1 Antitrypsin Inhibits ATP-Mediated Release of Interleukin-1β *via* CD36 and Nicotinic Acetylcholine Receptors

**DOI:** 10.3389/fimmu.2018.00877

**Published:** 2018-04-25

**Authors:** Kathrin Siebers, Bijan Fink, Anna Zakrzewicz, Alisa Agné, Katrin Richter, Sebastian Konzok, Andreas Hecker, Sven Zukunft, Mira Küllmar, Jochen Klein, J. Michael McIntosh, Thomas Timm, Katherina Sewald, Winfried Padberg, Nupur Aggarwal, Walee Chamulitrat, Sentot Santoso, Wendy Xia, Sabina Janciauskiene, Veronika Grau

**Affiliations:** ^1^Laboratory of Experimental Surgery, Department of General and Thoracic Surgery, Justus Liebig University Giessen, German Centre for Lung Research, Giessen, Germany; ^2^Fraunhofer Institute for Toxicology and Experimental Medicine, German Centre for Lung Research, Hannover, Germany; ^3^Institute of Vascular Signalling, Centre for Molecular Medicine, Goethe University, Frankfurt, Germany; ^4^Department of Pharmacology, Goethe University College of Pharmacy, Frankfurt, Germany; ^5^Department of Biology, University of Utah, Salt Lake City, UT, United States; ^6^George E. Wahlen Veterans Affairs Medical Center, Salt Lake City, UT, United States; ^7^Department of Psychiatry, University of Utah, Salt Lake City, UT, United States; ^8^Protein Analytics, Institute of Biochemistry, Justus Liebig University Giessen, Giessen, Germany; ^9^Department of Respiratory Medicine, Hannover Medical School, German Centre for Lung Research, Hannover, Germany; ^10^Department of Internal Medicine IV, University Heidelberg Hospital, Heidelberg, Germany; ^11^Institute for Clinical Immunology and Transfusion Medicine, Justus Liebig University Giessen, Giessen, Germany; ^12^Institute of Blood Transfusion, Guangzhou Blood Centre, Guangzhou, China

**Keywords:** CD36, CHRNA7, CHRNA9, CHRNA10, inflammasome, interleukin-1β, calcium-independent phospholipase A2β, P2X7 receptor

## Abstract

While interleukin (IL)-1β is a potent pro-inflammatory cytokine involved in host defense, high levels can cause life-threatening sterile inflammation including systemic inflammatory response syndrome. Hence, the control of IL-1β secretion is of outstanding biomedical importance. In response to a first inflammatory stimulus such as lipopolysaccharide, pro-IL-1β is synthesized as a cytoplasmic inactive pro-form. Extracellular ATP originating from injured cells is a prototypical second signal for inflammasome-dependent maturation and release of IL-1β. The human anti-protease alpha-1 antitrypsin (AAT) and IL-1β regulate each other *via* mechanisms that are only partially understood. Here, we demonstrate that physiological concentrations of AAT efficiently inhibit ATP-induced release of IL-1β from primary human blood mononuclear cells, monocytic U937 cells, and rat lung tissue, whereas ATP-independent IL-1β release is not impaired. Both, native and oxidized AAT are active, suggesting that the inhibition of IL-1β release is independent of the anti-elastase activity of AAT. Signaling of AAT in monocytic cells involves the lipid scavenger receptor CD36, calcium-independent phospholipase A2β, and the release of a small soluble mediator. This mediator leads to the activation of nicotinic acetylcholine receptors, which efficiently inhibit ATP-induced P2X7 receptor activation and inflammasome assembly. We suggest that AAT controls ATP-induced IL-1β release from human mononuclear blood cells by a novel triple-membrane-passing signaling pathway. This pathway may have clinical implications for the prevention of sterile pulmonary and systemic inflammation.

## Introduction

Interleukin (IL)-1β is a potent pro-inflammatory cytokine that plays a central role in host defense against infections and is mainly produced by monocytes, macrophages, and dendritic cells. Excessive systemic release of IL-1β, however, strongly contributes to the pathogenesis of life-threatening systemic inflammatory diseases including systemic inflammatory response syndrome (SIRS) (1). Release of IL-1β is strictly controlled and often depends on two consecutive danger signals. Lipopolysaccharide (LPS), a major cell wall component of Gram-negative bacteria, is a typical first signal that induces the biosynthesis of pro-IL-1β, an inactive cytoplasmic precursor of IL-1β. Numerous sterile and infectious signals can trigger the assembly of diverse multi-protein complexes, so-called inflammasomes that activate proteases to cleave pro-IL-1β (2). Extracellular ATP, mainly originating from the cytoplasm of damaged cells, is a typical second signal. ATP activates the purinergic receptor P2X7 (P2X7R) and enables an outward current of potassium ions (2). Reduced cytoplasmic potassium concentrations trigger the assembly of the NLRP3 (NACHT, LRR, and PYD domains-containing protein 3)-containing inflammasome, followed by the activation of caspase-1 (casp-1), cleavage of pro-IL-1β, and release of bioactive IL-1β (2). Mechanisms controlling ATP-induced inflammasome activation are in the focus of medical research because of their eminent relevance for the control of SIRS and sepsis.

Increasing evidence suggests that alpha-1 antitrypsin (AAT; synonym serine protease inhibitor, clade A, member 1, SERPINA1) interferes with the production of IL-1β and other pro-inflammatory cytokines (3–8), independent of its well-known function as a major inhibitor of proteases including neutrophil elastase (NE), proteinase 3, and chymase (4). During systemic inflammation, AAT synthesis is upregulated in the liver in response to circulating IL-6-type cytokines that are typically induced by IL-1β (4). Monocytes, macrophages, and dendritic cells are additional sources of AAT, albeit—in comparison to the liver—only small amounts are produced (9).

Under physiological conditions, plasma concentrations of AAT range from 0.9 to 2 mg/ml and a threefold to fourfold increase is typically measured during inflammation (4). Individuals with plasma AAT values below 0.7 mg/ml are considered to be AAT-deficient and have an increased risk for early-onset lung emphysema, liver disease and, in rare cases, vasculitis, asthma, and panniculitis (4). AAT infusions (e.g., Prolastin^®^) were approved for maintenance therapy of AAT-deficient patients suffering from emphysema and are generally well tolerated, but the efficiency of this treatment regarding emphysema is at best mediocre (10). By contrast, AAT therapy seems to be very effective in those AAT-deficient patients suffering from panniculitis, vasculitis, and asthma (5, 6), suggesting that AAT-mediated anti-inflammatory effects are most important.

Numerous studies on animal models of inflammatory diseases that involve IL-1β underscore the immuno-modulatory potential of AAT (5–7). AAT protects from experimental ischemia-reperfusion injury, rejection of islet allografts, graft-versus-host-disease, autoimmune arthritis, LPS-induced inflammation, and even exerts anti-bacterial effects (5–7). AAT has a strong, predominantly anti-inflammatory impact on the function of monocytes and neutrophils. It regulates chemotaxis, cell adhesion as well as the expression of toll-like receptor 4, chemokines, and pro-inflammatory cytokines. Furthermore, AAT reduces the production of superoxide and nitric oxide, and upregulates anti-inflammatory mediators (3, 4, 7). Nevertheless, the molecular mechanisms by which AAT modulates innate immunity are poorly understood.

Here, we test the hypothesis that ATP-dependent IL-1β release from blood leukocytes is regulated by AAT. We demonstrate that AAT activates a novel triple-membrane passing mechanism that inhibits ATP signaling and thereby, maturation and release of IL-1β by human blood leukocytes.

## Materials and Methods

### Reagents

Lipopolysaccharide (from *E. coli*, L2654), nigericin, BzATP, arachidonic acid (10931), cis-5,8,11,14,17-eicosapentaenoic acid (EPA, E2011), linoleic acid (LA, L1376), oleic acid (OA, O1008), mecamylamine hydrochloride, and strychnine hydrochloride were obtained from Sigma-Aldrich (Taufkirchen, Germany), thapsigargin, ATK, and BEL from Enzo Life Sciences (Lausen, Switzerland), α-bungarotoxin from Tocris Bioscience (Bristol, UK), and Prolastin^®^ from Grifols (Frankfurt, Germany). Conotoxins RgIA4 and ArIB were described previously (11–13). Polyclonal goat-anti-AAT was purchased from Bethyl (A80-122A, Montgomery, AL, USA), monoclonal rabbit anti-CD36 antibodies (clone D8L9T) from Cell Signaling Technologies (Danvers, MA, USA), and mouse anti-β-actin antibodies (clone A2228) from Sigma-Aldrich. Horseradish peroxidase-labeled secondary antibodies, rabbit anti-mouse Ig, rabbit anti-goat Ig, and goat anti-rabbit Ig, were provided by Dako (Glostrup, Denmark). The C-terminal peptide of AAT (C-36, corresponding to residues 359–394) was synthesized by JPT Peptide Technologies GmbH (Berlin, Germany) and provided with a purity of >95% (HPLC).

### Purification of AAT

Human plasma was obtained from healthy male non-smoking volunteers as approved by the local ethics committee of the University of Giessen (No. 81/13). AAT was isolated by affinity purification using Alpha1 Antitrypsin Select from GE Healthcare Europe (Freiburg, Germany) and Polyprep chromatography columns from Bio-Rad (Munich, Germany) essentially as described by the supplier followed by a buffer exchange to PBS using Amicon^®^ Ultra Centrifugal Filters with a cut-off of 10 kDa (Merck-Millipore, Darmstadt, Germany). The quality and efficiency of the purification was controlled by SDS-PAGE (7.5% acrylamide) and Brilliant Blue staining. To test for the anti-protease activity, purified AAT was incubated with NE (Merck-Millipore) at a 1:2 M ratio in 100 mM Tris/HCl pH 7.4 for 30 min at 37°C. Thereafter, the reaction was stopped by boiling in SDS-sample buffers and the samples were separated by SDS-PAGE.

### Oxidation of AAT

Oxidized AAT (oxAAT) was prepared as previously described (14). Briefly, *N*-chlorosuccinimide (Sigma-Aldrich) was added to AAT protein at a molar ratio of 25:1, incubated for 30 min at room temperature, and excess of *N*-chlorosuccinimide was removed by ultrafiltration. The quality of oxidation was controlled by testing anti-elastase activity. Native or oxAAT was pre-incubated with NE (at a molar ratio of 1.5:1) for 30 min. Thereafter, samples were analyzed by SDS-PAGE in 7.5% polyacrylamide gels followed by staining with Brilliant Blue.

### U937 Cells

U937 cells (German Collection of Microorganisms and Cell Cultures, Braunschweig, Germany) were kept in RPMI 1640 medium (Gibco, Thermo Fisher Scientific, Rockford, IL, USA) at 37°C and 5% CO_2_. Medium was supplemented with 10% fetal bovine serum (FBS Superior, Biochrom, Berlin, Germany) and 2 mM glutamin (Glutamax^®^, Gibco). Cells were primed with LPS (1 µg/ml) for 5 h and incubated with BzATP (100 µM) or nigericin (1–50 µM) for 30 min. In some experiments, nigericin was applied together with apyrase (0.5 U/ml, A6410, Sigma-Aldrich) to eliminate endogenous ATP. AAT and further reagents were applied together with BzATP. Supernatants were collected and stored at −20°C until measurement of IL-1β and lactate dehydrogenase (LDH).

### Human Leukocytes

The use of blood from healthy male non-smoking volunteers was approved by the local ethics committee of the University of Giessen (No. 81/13). Peripheral blood mononuclear cells (PBMC) were isolated by density gradient centrifugation using Leucosep gradients (Greiner Bio-One, Frickenhausen, Germany). CD14^+^ monocytes were enriched by positive selection using Dynabeads^®^ CD14 (Invitrogen, Karlsruhe, Germany) according to the instructions of the suppliers. Cell preparations were evaluated by flow cytometry (FACSCalibur^TM^, Becton Dickinson, San Jose, CA, USA) using FITC-labeled monoclonal antibody M5E2 to CD14 (BioLegend, San Diego, CA, USA). Monocyte purity was above 75%. Cells were cultured for 3 h and stimulated with BzATP as described previously (15) and AAT-P (1 mg/ml) was applied together with BzATP.

### Mouse PBMC

Breeding of calcium-independent phospholipase A2β (iPLA2β) gene-deficient mice (iPLA2β^−/−^) was performed at the animal facility of the University Heidelberg as described previously (16). Wild type (WT) C57/BL6 mice were purchased from Janvier Labs (Le Genest St. Isle, France). All experimental animals received humane care according to NIH “Guide for the Care and Use of Laboratory Animals.” Animal experiments were approved by the local committee at the Regierungspräsidium Giessen, Hesse, Germany (permit no. 571_M) and the Regierungspräsidium Karlsruhe, Baden-Württemberg, Germany (permit no. G248/11). PBMC isolation from female mice, culturing, and stimulation were performed as described previously (12).

### Rat Precision Cut Lung Slices (PCLS)

Female rats (Wistar WU, 10–12 weeks) were obtained from Charles River (Sulzfeld, Germany). Animals were kept under conventional housing conditions (22°C, 55% humidity, 12-h day/night rhythm). Rats were sacrificed by i.p. injection of an overdose of pentorbarbital sodium (Narkoren^®^, Merial GmbH, Hallbergmoos, Germany). Rat lungs were harvested, filled *ex situ* with 37°C warm 1.5% (wt/vol) low-melting agarose/DMEM solution and cooled in ice-cold DPBS after instillation. After preparation of cylindrical tissue cores (8 mm diameter), tissue was processed into approximately 300 µm thick slices with a Krumdieck tissue slicer (Alabama Research and Development, Munford, AL, USA). After washing of the slices, two PCLS were cultivated per well and stimulated with LPS (100 ng/ml) for 23.5 h. 30 min prior to collecting the supernatants, the slices were additionally treated with BzATP (150 µM). AAT-P (1 mg/ml) was applied together with BzATP.

### ELISA

Interleukin-1β concentrations were measured in cell culture supernatants by Human IL-1beta DuoSet (R&D systems, Minneapolis, MN, USA) combined with DuoSet ancillary kit (R&D systems) according to manufacturer’s instructions. Analogously, rat IL-1β concentrations were quantified in tissue culture supernatants by Rat IL-1β/IL-1F2 DuoSet (R&D systems, Minneapolis, MN, USA) and mouse IL-1β concentrations were measured in cell culture supernatants by using mouse Quantikine IL-1β Immunoassay (R&D Systems) according to manufacturer’s instructions. Measured cytokines released by PCLS were normalized against the total protein content of the tissue slices, assessed through a BCA protein assay kit (Pierce Biotechnology, Rockford, IL, USA).

### Cell Viability

Cell viability was assessed by measurement of LDH concentrations in cell culture supernatants. Supernatants were analyzed with CytoTox 96^®^ Non-Radioactive Cytotoxicity Assay (Promega, Madison, WI, USA). LDH release of *ex vivo* rat lung tissue was measured with a Cytotoxicity Detection Kit (Roche, Mannheim, Germany). Maximal LDH release was assessed by lysing the tissue slices with 1% Triton X-100 in DPBS (Lonza, Verviers, Belgium) at 4°C for 1 h.

### Gene Silencing

To silence the expression of CD36 and iPLA2β in U937 cells, Amaxa Cell Line Nucleofactor Kit C and Nucleofactor II Device (both from Lonza Cologne, Cologne, Germany) were used. Cells were transfected with ON-TARGETplus SMARTpool siRNA targeting human CD36 and iPLA2β (*CD36* and *PLA2G6*), as well as with Non-targeting Control Pool (Thermo Fisher Scientific, Schwerte, Germany) at a concentration of 30 pM siRNA/1 × 10^6^ cells.

### Co-Immunoprecipitation

Lipopolysaccharide-primed U937 cells (25 × 10^6^/ml) were lysed with agitation on ice for 30 min in 25 mM HEPES pH 7.2, 1 mM CaCl_2_, 1 mM MgCl_2_, 1% Triton-X100 supplemented with protease and phosphatase inhibitor (tablets from Thermo Fisher Scientific) and centrifuged. 500 µg AAT-P was loaded on 200 µl of Alpha1 Antitrypsin Select (GE Healthcare) incubated with the supernatant of the lysate over night at 4°C. In controls, AAT-P was omitted. After incubation, Alpha1 Antitrypsin Select beads were washed with lysis buffer, bound complexes were eluted as indicated by the supplier, mixed with concentrated SDS sample buffer, and subject to Western blotting.

### SDS-PAGE and Western Blotting

U937 cells were lysed and the protein concentration was assessed using a BCA protein assay kit (Pierce Biotechnology, Rockford, IL, USA). 10 µg of protein per sample was fractionated by SDS-PAGE on 10% gels and transferred to Immobilon polyvinylidene difluoride membranes (Millipore, Billerica). Dual color precision plus protein standards (Bio-Rad, Hercules, CA, USA) were used as molecular weight markers. Membranes were blocked with Roti^®^-Block (Roth, Karlsruhe, Germany) (CD36), 5% low-fat milk powder (Roth) (β-actin) or 5% BSA (AAT) in PBS and incubated with primary antibodies (anti-AAT 1:20,000, anti-CD36 1:1,000, β-actin 1:50,000) in blocking solution. PBS supplemented with 0.01% Tween-20 was used for washing steps and appropriate secondary antibodies (1:5,000 each) were diluted in PBS, 0.01% Tween-20, 2.5% low-fat milk powder. SuperSignal West Dura Extended Duration Substrate (Thermo Fisher Scientific) was used for the detection of CD36, blots were developed using Lumi-Light substrate (Roche, Mannheim, Germany) to visualize β-actin and documented with High Performance Chemiluminescence Films (GE Healthcare).

### Sera From Type I CD36-Deficient Patients

Sera from CD36 type I-deficient patients were collected from healthy blood donors, mothers with fetal/neonatal alloimmune thrombocytopenia and platelet transfusion refractoriness by Guangzhou Blood Center, Guangzhou, China. The presence of anti-CD36 antibodies in these sera and the absence of CD36 expression on monocytes and platelets were confirmed by flow cytometry and nucleotide sequencing analysis. The collection and the use of these sera for research were approved by Guangzhou Ethics Committee for human research (GZBC-EA-2016-010), and all patients gave written informed consent.

### Patch-Clamp Experiments

Whole-cell patch-clamp recordings on U937 cells where essentially performed as described previously (15). AAT-P and mecamylamine were used at concentrations of 1 mg/ml and 100 µM, respectively.

### Ultrafiltration of Cell Culture Supernatants

U937 cells were primed with LPS as described above but in the absence of FBS and stimulated with AAT-P (1 mg/ml). Thereafter, the cell-free supernatant was ultrafiltrated using Amicon^®^ Ultra Centrifugal Filters with a cut-off of 10 kDa. The resulting low molecular mass fraction was applied at different concentrations to another set of LPS-primed U937 cells shortly before stimulation with BzATP.

### Statistics

Statistical analyses were performed using SPPS^®^ (Version 23, IBM^®^, Armonk, NY, USA). Multiple data groups were first analyzed using Kruskal–Wallis test followed by two-tailed Mann–Whitney rank sum test. Data on primary cells were analyzed using Wilcoxon signed-rank test. IC_50_ values were determined using GraphPad Prism^®^ (Version 6, GaphPad Software) by fitting log-transformed concentration values and the original effect data.

## Results

### AAT Inhibits the ATP-Dependent Release of IL-1β

**Figure 1:**
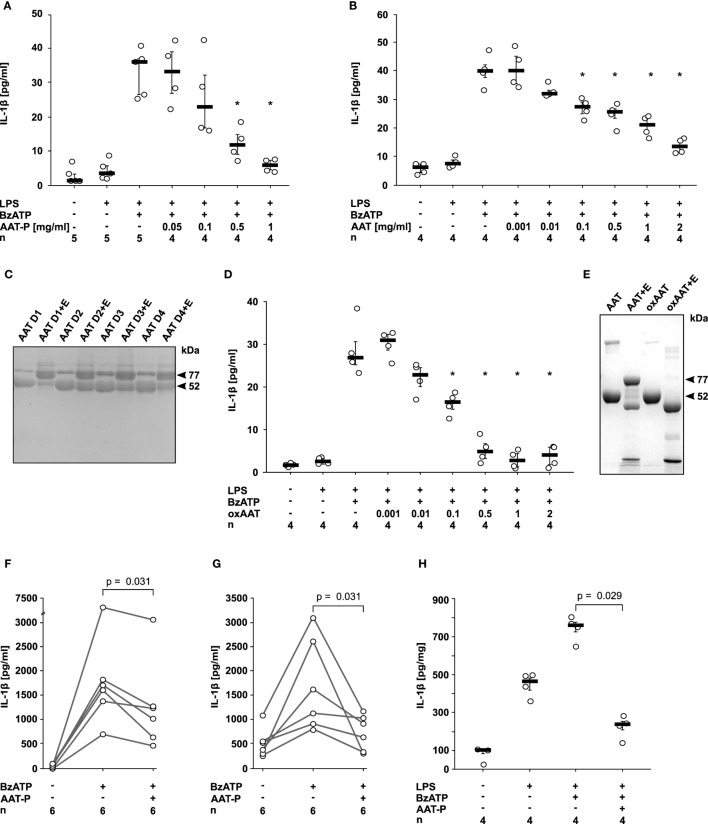
Native and oxidized alpha-1 antitrypsin (AAT) inhibit the ATP-dependent release of interleukin (IL)-1β. **(A,B,D)** Human lipopolysaccharide (LPS)-primed monocytic U937 cells were stimulated with 2′(3′)-*O*-(4-Benzoylbenzoyl)adenosine-5′-triphosphate (BzATP, 100 µM) in the presence or absence of different concentrations of the AAT preparation Prolastin^®^ [**(A)** AAT-P], of native AAT purified from the blood of healthy donors **(B)** and of oxidized AAT (oxAAT) **(D)**. IL-1β released to the supernatant was measured after 30 min. Native **(C)** and oxAAT **(E)** were separated by SDS-polyacrylamide gel electrophoresis (7.5% acrylamide) and stained with Brilliant Blue. In addition, native AAT from healthy donors (D1–D4) and oAAT were incubated with neutrophil elastase (NE) before electrophoresis. Arrows are pointing to NE (25 kDa), a fragment of AAT (48 kDa), AAT (52 kDa), oxAAT (52 kDa), and to a covalent complex of cleaved AAT and NE (77 kDa). **(F,G)** BzATP-mediated release of IL-1β from freshly isolated peripheral blood mononuclear cells **(F)** or enriched monocytes **(G)** from healthy human donors in the presence or absence of AAT-P (1 mg/ml). Absolute IL-1β values obtained from individual donors are connected by lines. **(H)** Rat precision cut lung slices were primed with LPS followed by application of BzATP in the presence or absence of AAT-P (1 mg/ml). Data are expressed as the concentration of IL-1β released per milligram lung tissue protein. Data are presented as individual data points, bar represents median, whiskers encompass the 25th to 75th percentile, *n*-numbers of independent experiments are indicated in the figure. Experimental groups were compared by Wilcoxon signed-rank test (**F,G**) or Kruskal–Wallis test followed by Mann–Whitney rank sum test **(A,B,D,H)** (*p ≤ 0.05 compared to supernatants from cells treated with LPS and BzATP alone).

Human monocytic U937 cells were primed with LPS for 5 h, followed by application of 2′(3′)-*O*-(4-Benzoylbenzoyl)adenosine-5′-triphosphate (BzATP), an analog of ATP that preferentially activates the P2X7R. In line with our hypothesis, the preparation of human plasma AAT (Prolastin^®^, AAT-P) efficiently and dose-dependently inhibited the expected BzATP-induced release of IL-1β to the cell culture supernatant (IC_50_ 0.2 mg/ml; Figure 1A). Of note, typical AAT blood concentrations of healthy humans (0.9–2 mg/ml) are sufficient to completely suppress IL-1β release from U937 cells. The inhibitory effect of AAT-P was corroborated with AAT isolated from the blood of healthy donors (IC_50_ 0.1 mg/ml; Figure 1B). SDS-gel electrophoresis of these AAT preparations revealed one major band with an apparent molecular mass of about 52 kDa (Figure 1C). The anti-protease activity of the AAT from healthy donors was confirmed by complex (77 kDa) formation with NE (Figure 1C). Previous studies have shown that anti-inflammatory functions of AAT can be independent of its anti-protease activity (7). In the same line, oxAAT, which is devoid of anti-elastase activity, efficiently inhibited BzATP-induced IL-1β release by LPS-primed U937 cells (IC_50_ 0.05 mg/ml; Figures 1D,E). Cell death as measured by LDH activity in the supernatant was not affected by BzATP or AAT in these and in all following experimental settings. We selected AAT-P at a concentration of 1 mg/ml for most experiments of this study.

Next, we investigated PMMC and enriched primary monocytes from healthy human volunteers, in which isolation and culture already induces pro-IL-1β expression (15). AAT-P (1 mg/ml) significantly (*p* = 0.031, *n* = 6 each) reduced the BzATP-induced release of IL-1β from these cells to about 50% (Figures 1F,G).

We studied LPS-primed rat PCLS, an established *ex vivo* model for pulmonary inflammation (17). AAT-P (1 mg/ml) fully inhibited the BzATP-induced IL-1β release (*p* = 0.029, *n* = 4, Figure 1H). We suggest that—in line with our hypothesis—AAT is a strong inhibitor of ATP-induced IL-1β release by human PBMC as well as by rat lung tissue.

### AAT Does Not Impair Nigericin-Dependent IL-1β Release

**Figure 2:**
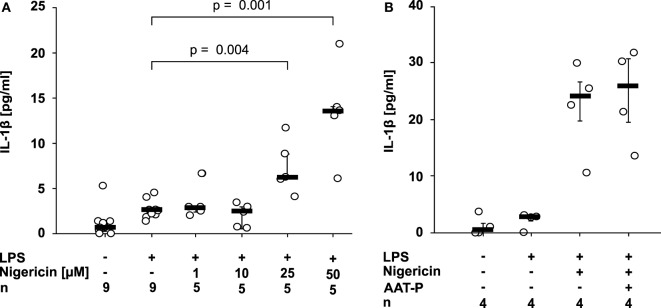
Alpha-1 antitrypsin (AAT) does not inhibit the nigericin-induced release of interleukin (IL)-1β. Human lipopolysaccharide (LPS)-primed monocytic U937 cells were stimulated with different concentrations of nigericin (1–50 µM) in the absence **(A)** or presence **(B)** of apyrase (0.5 U/ml) and of the AAT preparation Prolastin^®^ (AAT-P) (1 mg/ml). IL-1β released to the supernatant was measured after 30 min. Data are presented as individual data points, bar represents median, whiskers encompass the 25th to 75th percentile, *n*-numbers of independent experiments are indicated in the figure. Experimental groups were compared by Kruskal–Wallis test followed by Mann–Whitney rank sum test.

Nigericin is a bacterial pore-forming toxin that enables transmembrane potassium efflux, assembly of the NLRP3 inflammasome, and activation of casp-1 independent of ATP (2). 50 µM nigericin was needed to induce a significant release of IL-1β within 30 min of incubation (*p* = 0.029, *n* = 4) (Figure 2A). At all concentrations, the amount of LDH released to the supernatant was unchanged. To test if AAT inhibits ATP-independent pathways of NLRP3 inflammasome activation, LPS-primed U937 cells were stimulated with nigericin (50 µM). In these experiments, the ATP degrading enzyme apyrase (0.5 U/ml) was added. We rejected the hypothesis that AAT directly inhibits casp-1, as AAT-P (1 mg/ml) did not impair the nigericin-induced release of IL-1β from LPS-primed U937 cells (*n* = 4; Figure 2B).

### AAT Signals *via* CD36

**Figure 3:**
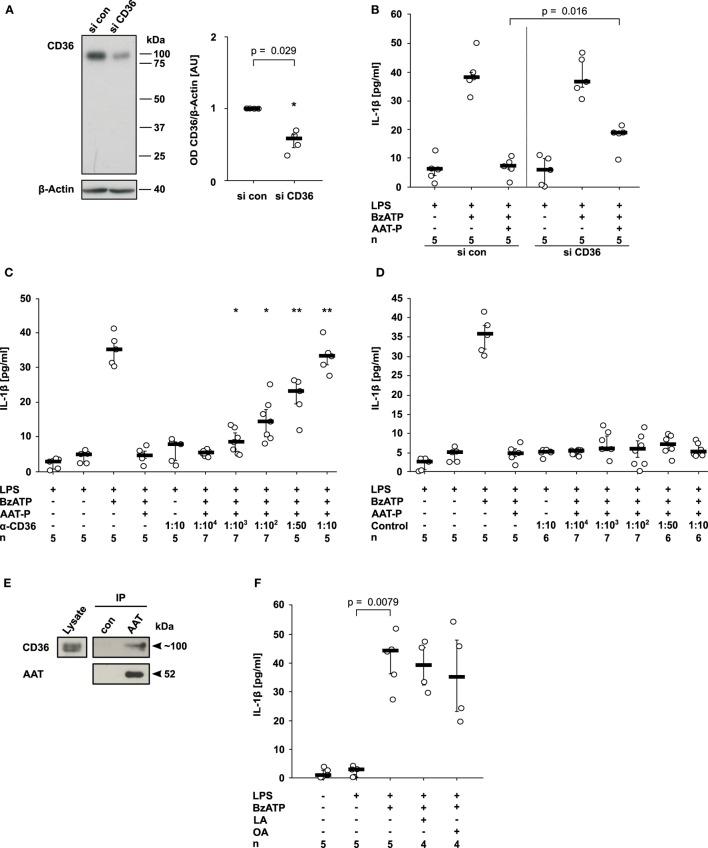
Alpha-1 antitrypsin (AAT) signaling involves CD36. **(A,B)** Expression of CD36 by U937 cells was silenced using siRNA (si CD36, *n* = 4). Another set of cells was transfected with control siRNA (si con, *n* = 4). **(A)** Cell extracts were separated by SDS-polyacrylamide gel electrophoresis (10% acrylamide) along with molecular weight markers. CD36 and β-actin protein expression were evaluated by Western blotting followed by densitometry. The ratio of the optical density (OD) of CD36- and β-actin-immunopositive bands was formed, normalized to the values obtained for si con-transfected cells and expressed as arbitrary units (AU). **(B)** U937 cells were transfected with si con or with si CD36, primed with lipopolysaccharide (LPS) and release of interleukin (IL)-1β was stimulated with 2′(3′)-*O*-(4-Benzoylbenzoyl)adenosine-5′-triphosphate (BzATP, 100 µM) in the presence or absence of the AAT preparation Prolastin^®^ (AAT-P, 1 mg/ml). **(C,D)** LPS-primed U937 cells were stimulated with BzATP in the presence of AAT-P and different concentrations of serum containing antibodies to CD36 **(C)** and control serum from healthy human donors **(D)**. **(E)** AAT (Prolastin^®^) was added to lysates of LPS-primed U937 cells and immunoprecipitated. Co-immunoprecipitation of CD36 was visualized Western blotting using antibodies to CD36, precipitation of AAT using antibodies to AAT. In controls (con), no AAT was added to the lysate. **(F)** LPS-primed U937 cells were stimulated with BzATP in the presence or absence of linoleic acid (LA, 2 mM) or oleic acid (OA, 2 mM). **(A–D,F)** Data are presented as individual data points, bar represents median, whiskers encompass the 25th to 75th percentile, *n*-numbers of independent experiments are indicated in the figure. Experimental groups were compared by Kruskal–Wallis where applicable **(B–D)**, followed by Mann–Whitney rank sum test **(A–D)** (*p ≤ 0.05; **p ≤ 0.01 compared to supernatants from cells treated with LPS, BzATP, and AAT-P).

CD36, a lipid scavenger receptor and taste receptor of long chain fatty acids (18), was shown before to interact with a fragment of AAT (19). Therefore, we studied its involvement in the signaling of AAT. CD36 expression was successfully silenced in U937 cells by siRNA (*n* = 4, *p* = 0.029; Figure 3A). In comparison to U937 cells treated with control siRNA, CD36 silencing significantly blunted the inhibitory effect of AAT-P on the BzATP-induced IL-1β release from LPS-primed U937 cells (*n* = 5, *p* = 0.016; Figure 3B). To corroborate the involvement of CD36 in AAT signaling, we used sera from type I CD36-deficient individuals who developed antibodies against CD36 during pregnancies or due to incompatible platelet transfusions. Recently, CD36 expression in these patients was carefully analyzed by flow cytometry, the CD36 genes were sequenced, and the presence of anti-CD36 antibodies in the serum was verified (20, 21). These sera dose-dependently antagonized the inhibitory effect of AAT-P, whereas sera from healthy blood donors did not (Figures 3C,D). Of note, neither control sera nor sera containing anti-CD36 antibodies provoked the release of IL-1β in the absence of BzATP (Figures 3C,D). In line with these results, co-immunoprecipitation experiments suggested that CD36 and AAT-P can physically interact (Figure 3E). In conclusion, our data are in line with the hypothesis that AAT-P signals *via* CD36 to inhibit BzATP-induced release of IL-1β.

Do other ligands of CD36 also inhibit the ATP-induced release of monocytic IL-1β? Previously, we demonstrated that phosphatidylserine, another ligand of CD36 (22) does not inhibit the release of IL-1β in the same experimental setting (23). In the same line, LA (2 mM, *n* = 4) and OA (2 mM, *n* = 4) did not impair the BzATP-induced release of IL-1β by LPS-primed U937 cells (Figure 3F). These free long chain fatty acids are also known ligands of CD36 (24). In addition, pilot experiments suggested that the CD36-ligands arachidonic acid (25) (0.1–1,000 µM, *n* = 2 each), EPA (24) (40 µM, *n* = 2), and C-36, the C-terminal peptide fragment of AAT (19) (1 µg/ml–2 mg/ml, *n* = 2 each), are also inactive in this experimental setting.

### AAT Signaling Activates iPLA2β

**Figure 4:**
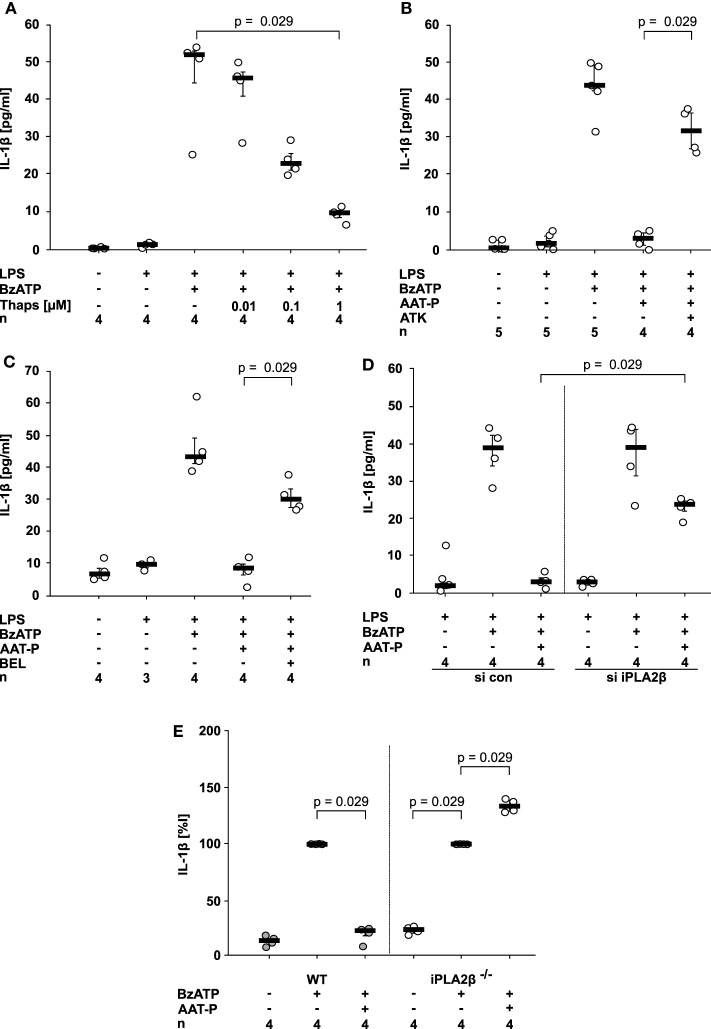
Alpha-1 antitrypsin (AAT) signaling involves activation of calcium-independent phospholipase A2β (iPLA2β). **(A–D)** Lipopolysaccharide (LPS)-primed U937 cells were stimulated with 2′(3′)-*O*-(4-Benzoylbenzoyl)adenosine-5′-triphosphate (BzATP) in the presence or absence of Prolastin^®^ (AAT-P, 1 mg/ml). Interleukin (IL)-1β released to the supernatant was measured after 30 min. Different doses of thapsigargin **(A)**, the unselective PLA2 inhibitor arachidonyl trifluoromethyl ketone [ATK, **(B)**] or the preferential iPLA2β inhibitor bromoenol lactone [BEL, **(C)**] were added together with BzATP and AAT-P. **(D)** Expression of iPLA2β by U937 cells was silenced by siRNA (si iPLA2β). In addition, cells were transfected with control siRNA (si con). U937 cells transfected with si con or with si iPLA2β were primed with LPS and the release of IL-1β was stimulated by BzATP. **(E)** Adherent peripheral blood mononuclear cells were isolated from wild type (WT) or iPLA2β gene-deficient mice (iPLA2β^−/−^) and stimulated with BzATP in the absence or presence of AAT-P. IL-1β released in response to BzATP was normalized to 100%. Data are presented as individual data points, bar represents median, whiskers encompass the 25th to 75th percentile, *n*-numbers of independent experiments are indicated in the figure. Experimental groups were compared by Kruskal–Wallis test followed by Mann–Whitney rank sum test.

Ligand binding to CD36 can induce activation of members of the phospholipase A2 (PLA2) family (18, 26). To test if PLA2 activation can inhibit IL-1β release from U937 cells, we first used thapsigargin, a blocker of sarcoplasmic-endoplasmic Ca^2+^-ATPase (SERCA) that activates PLA2 in the context of store-operated calcium reentry. In line with our hypothesis, thapsigargin dose-dependently and efficiently inhibited BzATP-induced release of IL-1β from LPS-primed U937 cells (Figure 4A).

This prompted us to investigate if PLA2 is involved in signaling of AAT-P. We used arachidonyl trifluoromethyl ketone (ATK, 50 µM) an inhibitor of both calcium-dependent PLA2 and calcium-independent PLA2 (iPLA2), as well as bromoenol lactone (BEL, 50 µM), a preferential inhibitor of iPLA2β (synonyms: group VIA iPLA2, iPLA2b, or PLA2G6). The inhibitory effect of AAT-P (1 mg/ml) on BzATP-induced IL-1β release was reversed by ATK (*n* = 4, *p* = 0.029; Figure 4B) and BEL (*n* = 4, *p* = 0.029; Figure 4C). We recently demonstrated in the same experimental setting that transfection of U937 cells with siRNA specific for iPLA2β resulted in a reduced expression of iPLA2β protein in comparison to cells transfected with control siRNA (27). Silencing of iPLA2β expression but not transfection of control siRNA significantly blunted the effect of AAT-P (1 mg/ml) in U937 cells (*n* = 4, *p* = 0.029; Figure 4D).

In an independent approach, we analyzed adherent PBMC from WT and iPLA2β gene-deficient mice (16). While AAT-P (1 mg/ml) efficiently inhibited BzATP-induced IL-1β release in WT PBMC, iPLA2β gene-deficient PBMC released a slightly higher amount of IL-1β in the presence of AAT-P (*n* = 4, *p* = 0.029; Figure 4E). Our results demonstrate that human AAT-P is equally active in mice and further support the hypothesis that iPLA2β is involved in signaling of AAT.

### AAT Signaling Involves Nicotinic Acetylcholine Receptors (nAChR)

**Figure 5:**
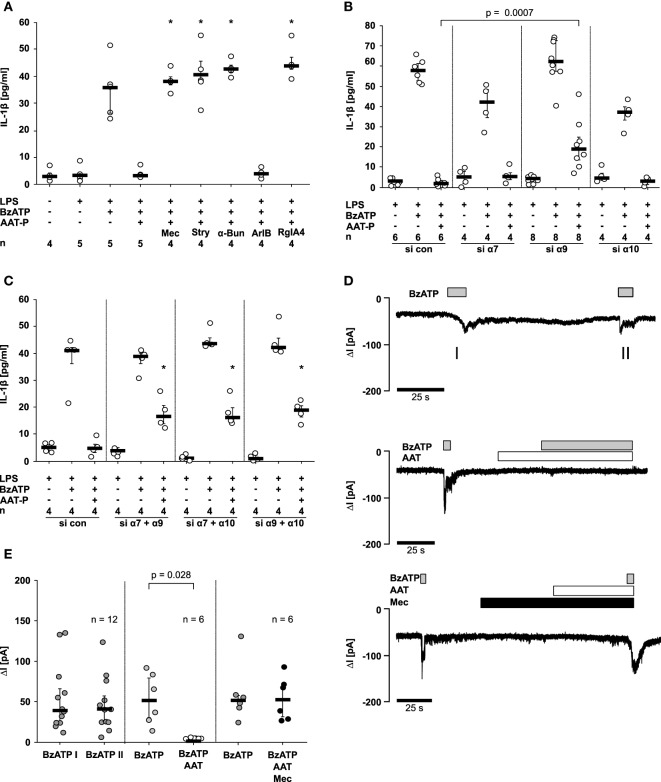
Alpha-1 antitrypsin (AAT) signaling involves nicotinic acetylcholine receptors (nAChR) and inhibits P2X7 receptor function. Lipopolysaccharide (LPS)-primed U937 cells were stimulated with 2′(3′)-*O*-(4-Benzoylbenzoyl)adenosine-5′-triphosphate (BzATP) in the presence or absence of Prolastin^®^ (AAT-P, 1 mg/ml). **(A–C)** Interleukin (IL)-1β released to the supernatant was measured after 30 min. **(A)** Nicotinic antagonists mecamylamine (Mec), α-bungarotoxin (α-Bun), strychnine (Stry), RgIA4, or ArIB [V11L, V16D] were added together with BzATP and AAT-P, *p ≤ 0.05 compared to cells treated with LPS, BzATP, and AAT-P. **(B,C)** Expression of nAChR subunits α7, α9, and α10 was silenced by siRNA (si α7, α9, α10). Cells were either transfected with a single siRNA **(B)** or with a combination of two of them **(C)**. In addition, cells were transfected with control siRNA (si con); *p ≤ 0.05, compared to data from respective experiments on cells treated with si con. **(C)**. **(D,E)** Whole-cell patch-clamp experiments were performed on LPS-primed U937 cells stimulated with BzATP. Patch-clamp recordings of U937 cells are depicted in **(D)**, and changes in ion currents (ΔI) are summarized in **(E)**. BzATP was applied twice (I and II). The second BzATP stimulus was given in the presence or absence of AAT-P and Mec. Data are presented as individual data points, bar represents median, whiskers encompass the 25th to 75th percentile, *n*-numbers of independent experiments are indicated in the figure. Experimental groups were compared by Wilcoxon signed-rank test **(E)** or by Kruskal–Wallis followed by Mann–Whitney rank sum test **(A–C)**.

Next, we investigated if AAT-P signals *via* nAChR using a set of nAChR antagonists at commonly used concentrations. Mecamylamine (100 µM), an unselective nicotinic antagonist, α-bungarotoxin (1 µM) and strychnine (10 µM), two antagonists of nAChR containing subunits α7 and α9, were applied to LPS-primed U937 cells shortly before application of AAT-P and BzATP. The inhibitory effect of AAT-P on the BzATP-induced release of IL-1β was sensitive to all three nicotinic antagonists (*n* = 4 versus 5, *p* = 0.016; Figure 5A). None of these antagonists induced IL-1β release when applied alone (data not shown). To differentiate between nAChR composed of subunits α7 and of α9α10, we used the α-conotoxin ArIB [V11L, V16D] (500 nM) that is a specific antagonist of receptors containing the α7 subunit and the α-conotoxin RgIA4 (200 nM), specific for α9 and α10 (11–13). ArIB was ineffective but RgIA4 fully reversed the effect of AAT-P (*n* = 4 versus 5, *p* = 0.016; Figure 5A), suggesting that α9 and/or α10 nAChR subunits are indispensable for the signaling of AAT.

To further dissect the involvement of nAChR subunits in AAT-P signaling, subunits α7, α9, and α10 in U937 cells were silenced by siRNA. Due to a lack of specific antibodies to nAChR subunits α7, α9, and α10, we could not monitor protein expression. The efficiency and specificity of gene-silencing in U937 cells was shown recently by our group in the same experimental setting for subunits α9 and α10 on the mRNA level (15). The mRNA expression of subunit α7, however, was too low for quantification. Transfection with siRNA targeting subunit α9 slightly but significantly impaired the effect of AAT-P on the BzATP-induced release of IL-1β (*n* = 6 versus *n* = 8, *p* = 0.0007), whereas single silencing of subunits α7 or α10 did not (Figure 5B). After double knockdown of combinations of α7, α9, or α10, AAT-P signaling was significantly blunted, in each receptor subunit combination (*n* = 4 each, *p* = 0.029; Figure 5C). These results suggest that nAChR subunits α7, α9, and α10 contribute to AAT-P signaling and that the concomitant function of at least two of them is mandatory. Single knockdown experiments suggest that subunit α9 might be more important than subunits α7 and α10.

### AAT Signaling Inhibits P2X7R

To test if the ATP-induced P2X7R activation is affected by AAT, patch-clamp experiments were performed on LPS-primed U937 cells. Application of BzATP induced ion currents, which were repeatable without obvious desensitization Figures 5D,E. BzATP was first applied alone, followed by application of AAT-P (1 mg/ml), which did not change ion currents by itself Figures 5D,E. When BzATP was applied in the presence of AAT-P, no changes of ion currents were detected (*n* = 6, *p* = 0.028; Figures 5D,E). The inhibitory effect of AAT-P on BzATP-induced ion channel function was fully antagonized in the presence of mecamylamine (100 µM) confirming the involvement of metabotropic nAChR functions in the AAT-P-mediated signaling (Figures 5D,E). These data are in line with the hypothesis that AAT-P inhibits BzATP-dependent P2X7R functions, an essential step in ATP-dependent inflammasome activation.

### AAT-P Triggers the Release of a Nicotinic Agonist

**Figure 6:**
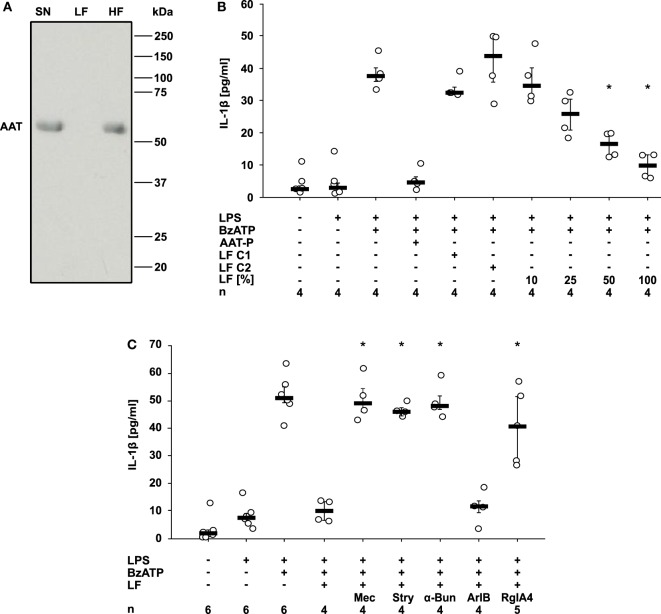
Alpha-1 antitrypsin (AAT) induces the release of a bioactive factor. U937 cells were primed with lipopolysaccharide (LPS) and stimulated with Prolastin^®^ (AAT-P 1 mg/ml) for 30 min before collection of the cell-free cell culture supernatant (SN). A low molecular mass fraction (LF) and a high molecular mass fraction (HF) of the cell culture supernatant were separated by ultrafiltration with a cut-off of 10 kDa. For the production of control LF, either no AAT-P was added to LPS-primed U937 cells (LF C1) or AAT-P was added to the cell-free cell culture supernatant (LF C2) shortly before ultrafiltration. **(A)** The SN, the LF, and the HF were separated in a 15% SDS-polyacrylamide gel along with a molecular mass marker followed by staining with Brilliant Blue. Bands with a molecular mass of about 52 kDa correspond to AAT. **(B,C)** LPS-primed U937 cells were stimulated with 2′(3′)-*O*-(4-Benzoylbenzoyl)adenosine-5′-triphosphate (BzATP) in the presence or absence of AAT-P. The experiments were performed in medium, LF C1, LF C2, or in different concentrations of LF diluted in medium. Interleukin (IL)-1β released to the supernatant was measured after 30 min. **(C)** Nicotinic receptor antagonists mecamylamine (Mec), α-bungarotoxin (α-Bun), strychnine (Stry), RgIA4, or ArIB [V11L, V16D] were added together with BzATP in the presence of LF. *p ≤ 0.05 versus cells treated with LPS, BzATP, and AAT-P **(B)** or LF **(C)**. Data are presented as individual data points, bar represents median, whiskers encompass the 25th to 75th percentile, *n*-numbers of independent experiments are indicated in the figure. Experimental groups were compared by Kruskal–Wallis followed by Mann–Whitney rank sum test **(B,C)**.

Finally, we wondered how AAT signaling *via* CD36 and iPLA2β links to the activation of nAChR. We demonstrated before that compounds with a phosphocholine head group including lysophosphatidylcholines function as unconventional nicotinic agonists that inhibit the ion channel function of P2X7R in human monocytic cells (12, 15, 23, 28). As iPLA2β has the capacity to cleave phosphatidylcholines to free fatty acids and lysophosphatidylcholines, we hypothesized that AAT-P triggers the release of a bioactive factor that mediates the cholinergic inhibition of P2X7R and IL-1β release. LPS-primed U937 cells were treated with AAT-P (1 mg/ml), the cell culture supernatant was harvested 30 min later and ultrafiltrated at a cut-off of 10 kDa to remove AAT-P with a molecular mass of about 52 kDa (Figure 6A). Control supernatant was harvested from LPS-primed U937 cells in the same way but in the absence of AAT-P (control 1), or AAT-P was added to the cell-free supernatant shortly before ultrafiltration (control 2). Low molecular weight fractions were applied to another set of LPS-primed U937 cells together with BzATP and the release of IL-1β was measured 30 min later. In agreement with our prediction, the low molecular weight fraction dose-dependently inhibited BzATP-induced IL-1β release, whereas this activity was absent in controls 1 and 2 (Figure 6B). The inhibitory activity was sensitive to the same panel of nAChR antagonists like AAT-P (*n* = 4 each, *p* = 0.029; Figures 5A and 6C). We conclude that a low molecular weight compound is released from U937 cells in response to AAT-P that activates nAChR of U937 cells.

## Discussion

**Figure 7:**
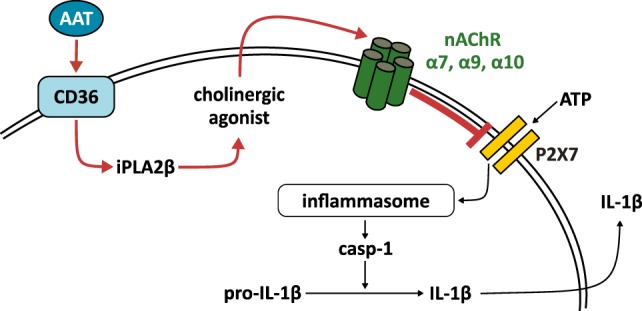
Suggested signaling mechanism of the alpha-1 antitrypsin (AAT)-induced control of ATP-induced inflammasome activation. Our results are in line with the following hypotheses: AAT binds to CD36 and induces activation of calcium-independent phospholipase A2β (iPLA2β), which cleaves membrane lipids and forms a yet unknown low molecular weight factor, presumably a cholinergic agonist. This agonist is released, activates nicotinic acetylcholine receptors (nAChR) containing subunits α9, α10, and/or α7 and inhibits the signaling of the ATP-sensitive P2X7 receptor. Our data suggest that AAT prevents inflammasome assembly, activation of caspase-1 (casp-1), and release of interleukin (IL)-1β. Red arrows symbolize mechanisms that were investigated in this study.

Alpha-1 antitrypsin is known to play an important protective anti-inflammatory role *in vivo* (3–7), but the underlying molecular mechanisms are poorly understood. Here, we demonstrate that physiological concentrations of AAT efficiently and dose-dependently inhibit the ATP-induced IL-1β release by monocytic U937 cells, PBMC, and lung tissue. The commercially available AAT preparation Prolastin^®^, AAT freshly isolated from the blood of healthy donors and oxAAT are active. We provide evidence for a triple-membrane passing signaling mechanism of AAT that is summarized in Figure 7. AAT seems to signal *via* CD36, followed by activation of iPLA2β and release of a low molecular weight factor with cholinergic activity. Stimulation of nAChR, in turn, inhibits the ATP-gated ion channel function of the P2X7R and, hence, ATP-induced maturation and release of IL-1β. The experimental settings used in this study induce IL-1β secretion independent of cell death, as the LDH content of the cell culture medium was not increased. It was shown before that under these conditions, U937 cells and primary human blood leukocytes do not release IL-18 (15, 29).

Most experiments of this study were performed on human U937 cells, a monocytic cell line that produces only low amounts of IL-1β (12, 15, 23, 27, 28). As cell lines do not reflect all properties of primary cells, we included several well-validated primary cell and tissue culture models for inflammation. Experiments on human PBMC, enriched human monocytes, mouse PBMC, and rat PCLS clearly confirmed the data obtained from U937 cells. We conclude that the AAT-mediated inhibition of ATP-induced IL-1β is active in humans, mice, and rats. An efficient control of the release of monocytic IL-1β into the circulation seems to be mandatory, as cytokines are rapidly swept away from the site of inflammation and may cause life-threatening systemic inflammation. Because of the prominent role of AAT in the lung (4), we analyzed PCLS and demonstrated that AAT fully prevents the ATP-induced release of pulmonary IL-1β. The control of pulmonary inflammation is of outstanding importance, because on the delicate lung tissue is constantly exposed to environmental noxes that have to be efficiently cleared without inducing overt inflammation (30–32).

Exogenous AAT can enter the cytoplasm *via* lipid rafts (33) and was suggested to directly inhibit the proteolytic activity of casp-1 (34, 35). Although the inhibition of cytoplasmic casp-1 of intact cells by AAT was disputed (36), we wondered if this mechanism is responsible for the AAT-mediated suppression of IL-1β release. This hypothesis was falsified by two independent experiments. First, IL-1β release triggered by nigericin, a pore-forming bacterial toxin that stimulates casp-1 activation in an ATP-independent manner (37), is not impaired. Second, we demonstrated in patch-clamp experiments that AAT inhibits the ion channel function of the P2X7R, a mechanism that is upstream of NLRP3 inflammasome assembly and casp-1 activation (2).

A report on interactions of CD36 with the fibril-forming C-terminal C-36 fragment (C-36) of AAT prompted us to hypothesize that CD36 is involved in AAT signaling (19). Indeed, CD36 gene-silencing significantly blunts the effect of AAT in our experimental setting. In the same line, sera from CD36-deficient patients who were sensitized to CD36 by blood transfusion or pregnancy (20, 21) dose-dependently reverse the inhibitory effect of AAT on the BzATP-induced release of IL-1β by U937 cells, while control sera from healthy donors do not. In the same line, immunoprecipitation experiments suggested a physical interaction of CD36 and AAT in LPS-primed U937 cells. We suggest that CD36 is an essential part of the signaling cascade induced by AAT and that CD36 might function as a cellular receptor for AAT.

Our results seem to contradict studies that suggest a pro-inflammatory role of CD36 (19, 38). CD36 contributes to cell priming *via* Toll-like receptors 4 and 6 and to inflammasome activation by promoting the cellular uptake of particle-forming matter such as oxidized LDL or amyloid-β (38). C-36 fragments interacting with CD36 and LDL receptors further stimulate of the uptake of oxidized LDL and induce the expression of pro-inflammatory cytokines IL-6 and monocyte chemoattractant protein-1 (19). As CD36 is a versatile molecule that can function as a receptor mediating signal transduction or as a transmembrane transporter, the down-stream effects of CD36 may be very different depending on the properties of the ligand and on the cell type investigated (25). In addition, we describe short-term effects (less than 30 min) of CD36 activation with AAT, whereas CD36-mediated expression of pro-inflammatory cytokines and inflammasome activation takes several hours (19, 38).

We reported before that the CD36 ligand phosphatidylserine does not inhibit the BzATP-induced release of IL-1β by LPS-primed U937 cells (22, 23), and we provide similar data in the present study for LA and OA, other ligands of CD36 (25). These results suggest that the described down-stream effects leading to the inhibition of IL-1β release are rather specific for AAT and cannot be elicited by all known ligands of CD36. CD36 exerts numerous cell-specific functions as a lipid transporter, as a scavenger receptor and as a taste receptor for free long chain fatty acids (25). Accordingly, different isoforms of CD36 have been described that differ in posttranslational modifications such a differential glycosylation (25). With all due caution, we suggest that monocytic CD36 functions as a receptor of AAT.

As signal transduction *via* CD36 typically activates iPLA2β, a phospholipase that hydrolyzes the sn-2 acyl bond of phosphatidylcholines (18, 26), we hypothesized that activation of iPLA2β is an essential step in AAT signaling. In a first proof-of-principle experiment, we demonstrated that thapsigargin, an inhibitor of Ca^2+^ reuptake by the endoplasmic reticulum and activator of iPLA2β (39), efficiently and dose-dependently inhibits the ATP-induced IL-1β release from LPS-primed U937 cells. We demonstrate that the general PLA2 inhibitor ATK and the more specific iPLA2 inhibitor BEL fully abolish the effect of AAT on IL-1β release and gene-silencing results in a significantly blunted effect of AAT. Furthermore, PBMC from iPLA2β gene-deficient mice are insensitive to AAT, whereas AAT almost fully abolishes the ATP-induced release of IL-1β by WT PBMC. We conclude from these data that iPLA2β is involved in the AAT-dependent signaling cascade that inhibits the ion channel function of P2X7R. A similar involvement of iPLA2β in the chemokine-dependent inhibition of IL-1β release was recently published by our group (27).

Activation of nAChR containing subunits α7, α9, and α10 with canonical ligands such as acetylcholine or nicotine efficiently suppresses BzATP-dependent ion currents and release of IL-1β from human monocytic cells (12, 15). We further identified phosphatidylcholines as well as their metabolites lysophosphatidylcholine, glyceophosphocholine, and phosphocholine as unconventional agonists of nAChR. These agonists inhibit P2X7R signaling in human monocytic cells but do not evoke ion currents at conventional nAChR (12, 23, 28). Interestingly, these unconventional nicotinic agonists slightly differed in their requirements for nAChR subunits. Signaling of phosphocholine depends on subunits α7, α9, and α10 (12, 15, 28), lysophosphatidylcholine and glycerophosphocholine depend on subunits α9 and α10, and dipalmitoylphosphatidylcholine depends on subunit α9 in combination with either subunit α7 or α10 (23, 28). As iPLA2β cleaves phosphatidylcholines and liberates lysophosphatidylcholines that can function as nicotinic agonists (28), we hypothesized that AAT induces the release of a factor that acts as an unconventional agonist of nAChR.

Signaling of AAT is indeed sensitive to the nictonic antagonists mecamylamine, strychnine, and α-bungarotoxin, suggesting that the evolutionary conserved family of nAChR comprising subunits α7, α9, and α10 is involved (40–42). Among them, subunits α9 or α10 seem to be mandatory, because the conotoxin RgIA4 fully inhibits the effect of AAT, while conotoxin ArIB is ineffective, suggesting that subunit α7 is dispensable (11–13, 43). The importance of subunit α9 is corroborated in gene-silencing experiments, whereas single knockdown of subunits α7 or α10 has no effect. All combinations of double-knockdowns, however, lead to a blunted inhibitory effect of AAT. Taken together, nAChR subunit α9 seems to be mandatory but not sufficient for AAT signaling. In addition, either subunit α7 or subunit α10 are needed.

In line with numerous previous studies showing that nicotinic stimuli do not induce ion channel functions at leukocytic nAChR (12, 15, 44–48), AAT does not trigger ion currents in LPS-primed U937 cells. By contrast, BzATP provokes repeatable ion currents that are prevented in the presence of AAT. Also in this experimental setting, the effects of AAT are sensitive to mecamylamine. These data suggest that much like nicotine, phosphocholine, glycerophosphocholine, palmitoyl-lysophosphatidylcholine, or dipalmitoylphosphatidylcholine (12, 15, 23, 28), AAT activates nAChR that inhibit the ion channel function of P2X7R.

Finally, we show that LPS-primed U937 cells release small bioactive molecules in response to AAT that efficiently and dose-dependently inhibit the BzATP-induced IL-1β release. The activity of these molecules is reversed by nAChR antagonists of subunits α9/α10 but not by the conotoxin ArIB, which is specific for subunit α7 (11–13). This sensitivity toward nAChR antagonists is the same for both, AAT and the small molecular weight factor released in response to AAT. Hence, we suggest that AAT induces the release of nicotinic agonists that mediate the activation of nAChR. We are currently trying to elucidate the chemical identity of the bioactive factor(s) that are released in response to AAT. As iPLA2β is involved in signaling, metabolites of phosphatidylcholines are plausible candidates. The free long-chain fatty acids tested in this study were inactive, but all compounds with a phosphocholine head group we investigated inhibited the BzATP-induced release of IL-1β (12, 15, 28). Therefore, we hypothesize that the active mediators are phosphocholine-containing metabolites of phosphatidylcholines, but these are numerous and diverse.

Our current working hypothesis of the signaling cascade of AAT is depicted in Figure 7. We suggest a triple-membrane-passing signaling pathway triggered by AAT that involves activation of CD36, iPLA2β-dependent release of a low molecular weight factor. This factor activates metabotropic nAChR receptor functions that inhibit the P2X7R as well as activation and release of IL-1β.

A limitation of our study is that we only describe the general lines of a signaling cascade that is probably more complex. For instance, the exact mechanism of iPLA2β activation is unknown as well as the substrates of this phospholipase. We can only speculate on the nature of the released low molecular weight factor and even do not know if it is a single active chemical substance or a complex mixture. Similarly, metabotropic signaling mechanisms of nAChR are an emerging field of research and have to be analyzed in detail (48, 49). In addition, we only provide *in vitro* and *ex vivo* evidence for the control of IL-1β release. More preclinical and clinical research is warranted to estimate the biological and medical relevance of this mechanism.

The strength of our study is that we provide evidence for a very efficient pathway by which physiological concentrations of AAT inhibit the ATP-induced release of IL-1β by human monocytic cells. Our findings might mechanistically explain recent data on a large cohort of healthy individuals, in whom a negative correlation of AAT and IL-1β blood levels was observed (50). Extracellular ATP is a danger molecule and trigger for NLRP3 inflammasome activation that is mainly released from the cytoplasm of damaged cells. ATP-induced IL-1β release only plays a subordinate role in during infection, where other inflammasomes and other mechanisms of caspase activation dominate (2). Hence, we provide first evidence for an AAT-mechanism that has the potential to protect against sterile, trauma-associated SIRS, without preventing host defense against infections. In addition, we discovered a triple-membrane-spanning signal transduction mechanism involving CD36, iPLA2β, and nAChR that might regulate other functions in immunity and beyond.

Our results suggest that physiological concentrations of AAT might act as an endogenous safeguard against overshooting inflammation mediated by extracellular ATP. The potential clinical use of AAT for the prevention of trauma-associated SIRS deserves further investigation.

## Ethics Statement

The collection and the use of these sera for research were approved by the Ethics Committee of the University of Giessen (No. 81/13) and by the Guangzhou Ethics Committee for human research (GZBC-EA-2016-010), and all patients gave written informed consent.

## Author Contributions

KSi, AZ, KR, JK, JM, KS, WP, NA, WC, SS, SJ, and VG contributed to research design and to the interpretation of the data; KSi, BF, AZ, AA, KR, SK, AH, SZ, MK, TT, KSe, and WX contributed to the performance of experiments; VG wrote the manuscript; all authors read and edited the manuscript.

## Conflict of Interest Statement

Certain conotoxins, including RgIA4 have been patented by the University of Utah; JM is an inventor on these patents. All other authors declare that no conflict of interest exists.
